# Supplementary material for: Structure dynamics of HIV-1 Env trimers on native virions engaged with living T cells
Source: Commun Biol. 2021 Oct 27;4:1228. doi: 10.1038/s42003-021-02658-1 (PMC8551276; doi:10.1038/s42003-021-02658-1)
Supplement: Supplementary file 2 — Supplementary Information [file 42003_2021_2658_MOESM2_ESM.pdf]

SUPPLEMENTARY INFORMATION

Structure dynamics of HIV-1 Env Trimers on Native virions Engaged with Living T Cells

Irene Carlon-Andres[1,2,3*], Tomas Malinauskas[3], Sergi Padilla-Parra[1,2,3]*.

Affiliations:

[1]Department of Infectious Diseases, King's College London, Faculty of Life Sciences & Medicine, London SE1 9RT, UK

[2]Randall Division of Cell and Molecular Biophysics, King's College London, London SE1 9RT, UK.

[3]Division of Structural Biology, Wellcome Centre for Human Genetics, University of Oxford, Oxford OX3 7BN, UK

*Lead Contact: irene.carlon-andres@kcl.ac.uk, sergio.padilla_parra@kcl.ac.uk

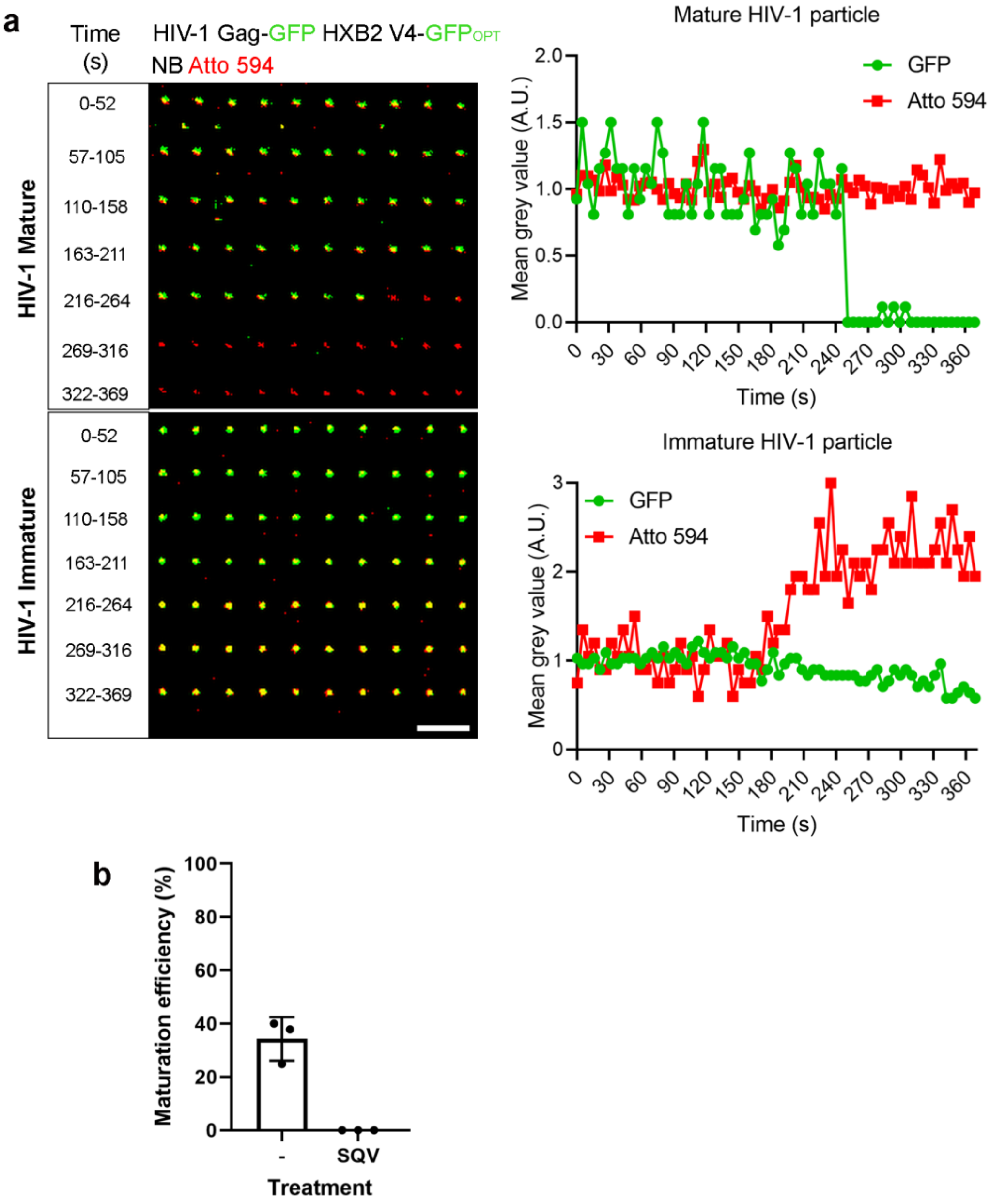

Supplementary Figure 1. Labelling and maturation efficiency of HXB2 V4-GFP$_{OPT}$ virions. a Single particle tracking of mature and immature virions upon saponin-induced membrane permeabilization. The micrograph shows a double labelled HIV-1 mature particle (GFP+ Atto 594+) releasing the GFP content at ~240 s after saponin addition, as observed by a drop in green fluorescence intensity. Membrane permeabilization in immature HIV-1 particles instead allows access to uncleaved Gag-GFP by NbA594, judged by an increase in red fluorescent intensity at ~200 s after saponin addition. Scale bar 5 µm. A.U.: arbitrary units. **b** Bar graph showing the mean and SD of the relative number of mature and immature virions of n=3 viral samples prepared independently in absence (-) or presence of saquinavir (SQV) treatment. At least 100 viral particles were analyzed per condition, per experiment.

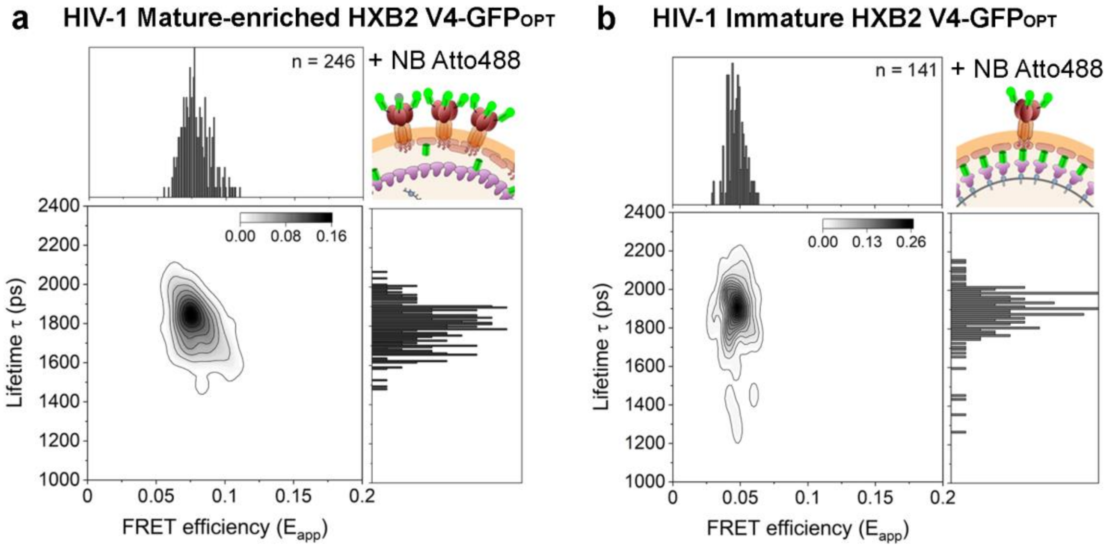

Supplementary Figure 2. FRET negative controls for intramolecular and intermolecular HIV-1 Env conformations. Two-dimensional (2D) kernel probability graphs showing FRET (FRET efficiency, E_{app}) vs FLIM (Lifetime, in ps) data. **a** Mature-enriched or **b** Immature HXB2 V4-GFP$_{OPT}$ bearing Gag-GFP labelled with NbA488. Absence of acceptor delimits the no-FRET threshold of $E_{app} = 0.1$ for mature-enriched viral sample and $E_{app} = 0.07$ for immature particles.

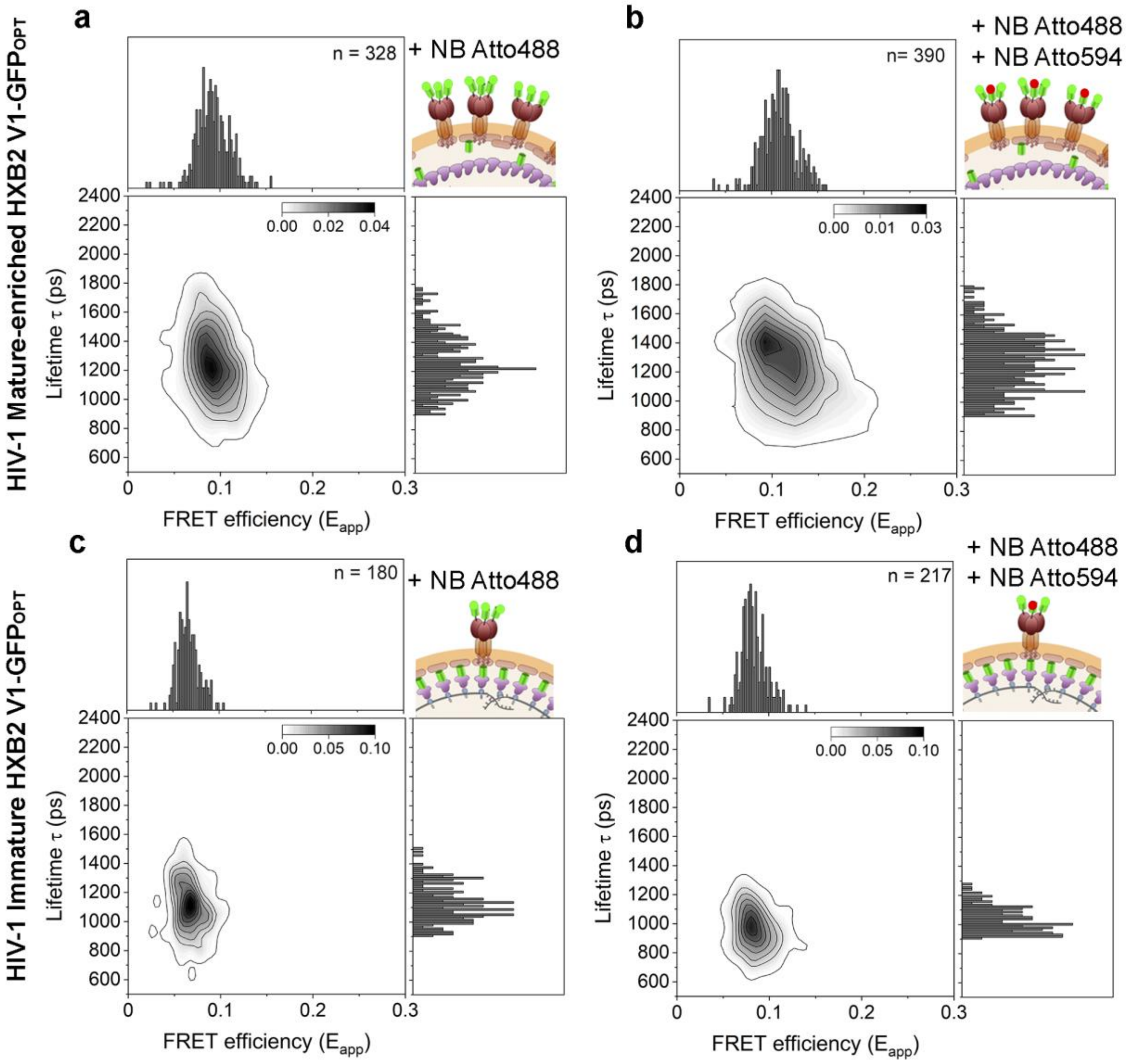

Supplementary Figure 3. FRET negative controls for intramolecular and intermolecular HIV-1 Env conformations. Two-dimensional (2D) kernel probability graphs showing FRET (FRET efficiency, E_{app}) vs FLIM (Lifetime, in ps) data. (**a-b**) Mature-enriched sample of HXB2 V4-GFP_{OPT} bearing Gag-GFP labelled with the donor, NbA488 (**a**) or double labelled, NbA488 and NbA594 (**b**). (**c-d**) Immature sample of HXB2 V4-GFP_{OPT} bearing Gag-GFP labelled with the donor, NbA488 (**c**) or double labelled, NbA488 and NbA594 (**d**). Addition of the acceptor NbA594 in presence of the donor fluorophore NbA488 induces a shift towards higher FRET efficiencies in both, (**b**) mature and (**d**) immature particles below 0.23, which determines the threshold for intermolecular interactions observed in gp120 V4-labelled virions.

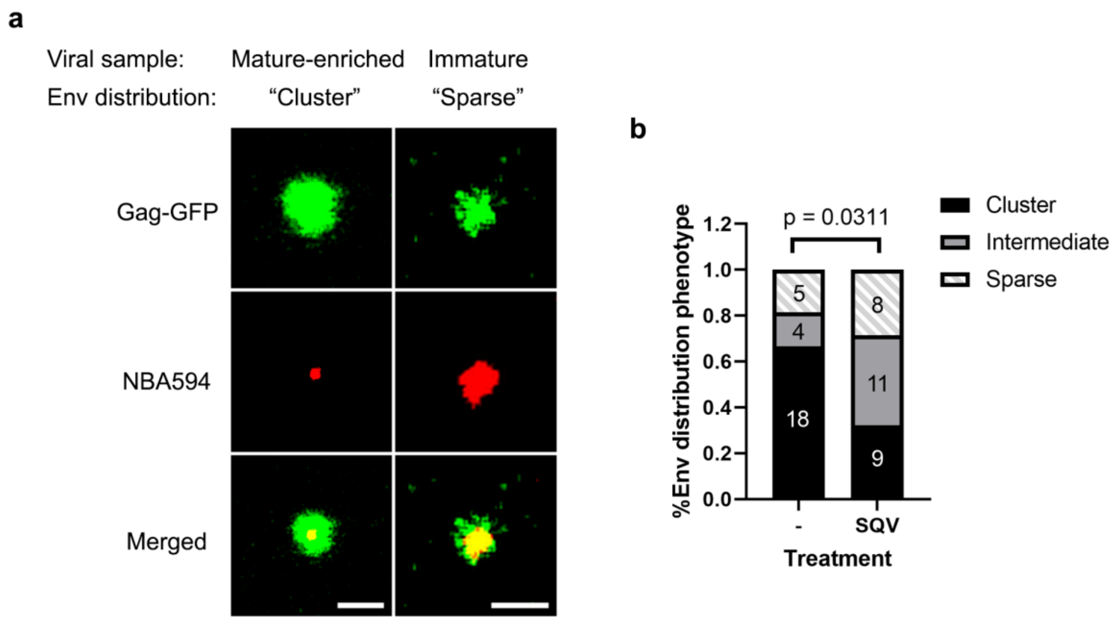

Supplementary Figure 4. STED microscopy shows different HXB2-GFP$_{OPT}$-Nb594 distribution patterns for mature and immature viruses. a Representative STED images of Gag-GFP (green) and HXB2-GFP$_{OPT}$ labelled with NbA594 (red) for Env "cluster" phenotype predominantly found in mature-enriched samples and Env "sparse" distribution observed in immature HIV-1 particles. Scale bar: 500 nm. **b** Env distribution patterns were recovered by drawing a plot profile to recover the Env signal distribution around the virus particles. Three different patterns with different peaks and distributions were recovered. Particles were represented statistically in a bar diagram representing the occurrence of each phenotype for Env distribution (cluster, intermediate, sparse) and significance was assessed by $\chi 2$ test. Absolute numbers of viral particles analyzed are indicated on the bars for each distribution pattern, per condition.

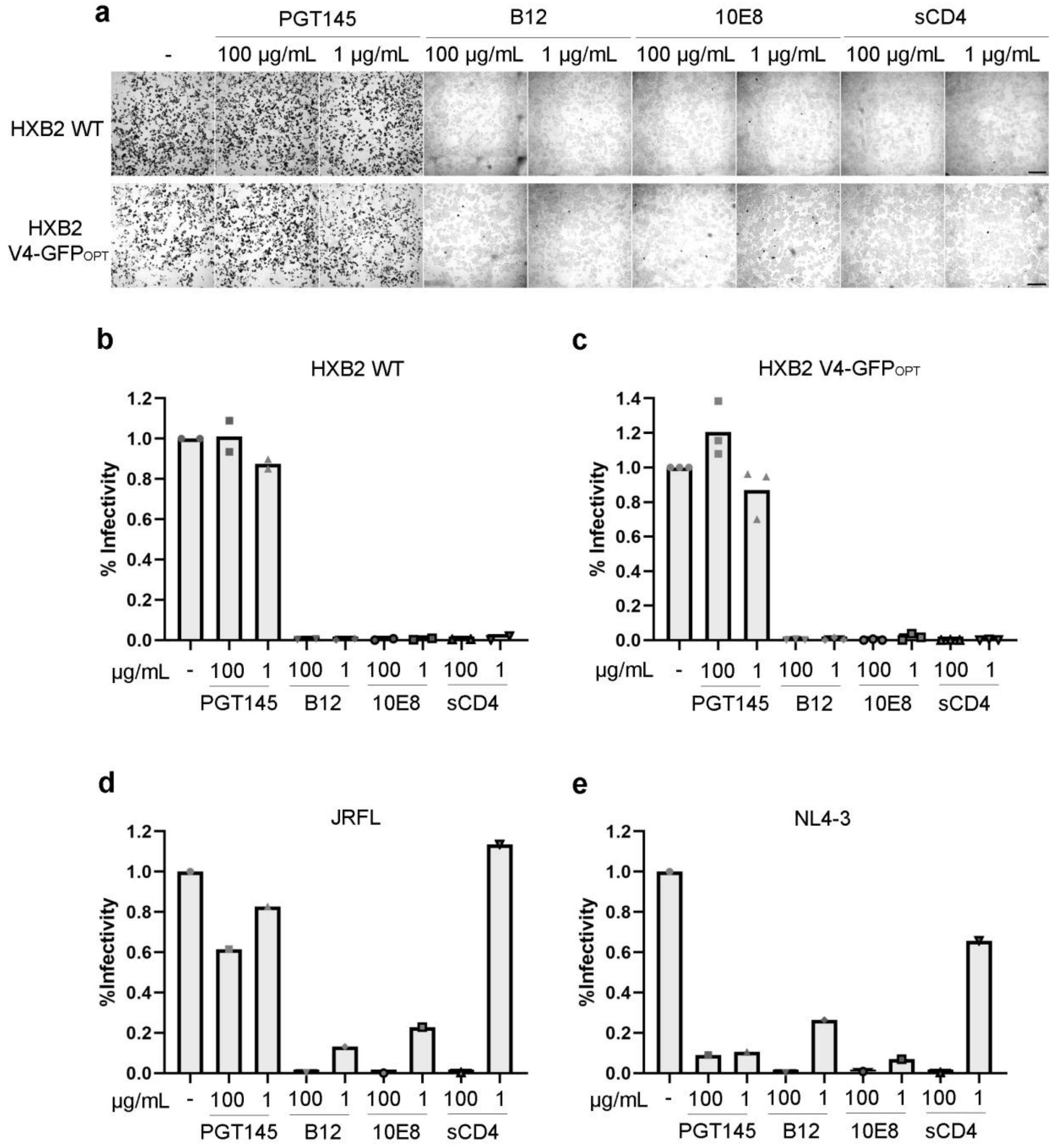

Supplementary Figure 5. Neutralization efficiency of bNAbs on different HIV-1 strains. TZM-bl cells were infected with equivalent amounts of pseudo-typed HIV-1 virus in absence (-) or presence of PGT145, b12, 10E8 antibodies or sCD4 (100 µg/mL or 1 µg/mL, as indicated). **a** Micrograph of transmission light microscopy showing infection efficiency of HXB2 WT pseudo-typed viruses (upper row) or HXB2 V4-GFP$_{OPT}$ (bottom row) in absence or presence of different ligands. Scale bar 0.5 mm. (**b-c**) Column graphs represent the mean and dots individual values showing the relative sensibility to antibody or receptor binding of (**b**) HXB2 WT pseudo-typed viruses (n=2 independent experiments) or (**c**) HXB2 V4-GFP$_{OPT}$ pseudo-typed viruses (n=3 independent experiments). (**d-e**) Column graphs represent the relative sensibility to antibody or receptor binding of (**d**) JRFL pseudo-typed viruses or (**e**) NL4-3 pseudo-typed viruses.
